# Relation of 24-hour urinary caffeine and caffeine metabolite excretions with self-reported consumption of coffee and other caffeinated beverages in the general population

**DOI:** 10.1186/s12986-016-0144-4

**Published:** 2016-11-17

**Authors:** Dusan Petrovic, Sandrine Estoppey Younes, Menno Pruijm, Belén Ponte, Daniel Ackermann, Georg Ehret, Nicolas Ansermot, Markus Mohaupt, Fred Paccaud, Bruno Vogt, Antoinette Pechère-Bertschi, Pierre-Yves Martin, Michel Burnier, Chin B. Eap, Murielle Bochud, Idris Guessous

**Affiliations:** 1 Institute of Social and Preventive Medicine (IUMSP), Lausanne University Hospital, Route de la corniche 10, Lausanne, 1010 Switzerland; 2 Department of Nephrology and Hypertension, Lausanne University Hospital, Rue du Bugnon 17, Lausanne, 1011 Switzerland; 3 Department of Nephrology and Hypertension, University Hospital of Geneva (HUG), Rue Gabrielle Perret-Gentil 4, Geneva, 1205 Switzerland; 4 University Clinic for Nephrology, Hypertension and Clinical Pharmacology, Inselspital, Bern University Hospital, University of Bern, Freiburgstrasse 15, Bern, 3010 Switzerland; 5 Department of Cardiology, University Hospital of Geneva (HUG), Rue Gabrielle Perret-Gentil 4, Geneva, 1205 Switzerland; 6 Unit of Pharmacogenetics and Clinical Psychopharmacology, Centre for Psychiatric Neuroscience, Department of Psychiatry, Lausanne University Hospital, Prilly, 1008 Switzerland; 7 School of Pharmaceutical Sciences, University of Geneva, University of Lausanne, Geneva, Switzerland; 8 Unit of Population Epidemiology, Division of Primary Care Medicine, Department of Community Medicine and Primary Care and Emergency Medicine, University Hospital of Geneva (HUG), Rue Gabrielle Perret-Gentil 4, Geneva, 1205 Switzerland; 9 Department of Epidemiology, Rollins School of Public Health, Emory University, Atlanta, USA; 10 Lausanne University Outpatient Clinic, Rue du Bugnon 44, Lausanne, 1011 Switzerland; 11 Unit of Population Epidemiology, University Hospital of Geneva (HUG), Rue Gabrielle Perret-Gentil 4, Geneva, 1205 Switzerland

**Keywords:** Caffeine, Paraxanthine, Theophylline, Urinary excretion, Questionnaire, Population-based

## Abstract

**Background:**

Caffeine intake is generally estimated by self-reported consumption, but it remains unclear how well self-report associates with metabolite urinary excretion. We investigated the associations of self-reported consumption of caffeinated drinks with urinary excretion of caffeine and its major metabolites in an adult population.

**Methods:**

We used data from the population-based Swiss Kidney Project on Genes in Hypertension (SKIPOGH) study. Consumption of caffeinated coffee, decaffeinated coffee and other caffeinated beverages was assessed by self-administered questionnaire. Quantification of caffeine, paraxanthine, theobromine and theophylline was performed by ultra-high performance liquid chromatography tandem mass spectrometry in 24-h urine. Association of reported consumption of caffeinated drinks with urinary caffeine derived metabolites was determined by quantile regression. We then explored the association between urinary metabolite excretion and dichotomized weekly consumption frequency of caffeinated coffee, with Receiver Operator Characteristic (ROC) analysis.

**Results:**

In the present analysis, we included 598 individuals (52% women, mean age =46 ± 17 years). Self-reported caffeinated coffee intake was positively associated with 24-h urinary excretions of paraxanthine, theophylline and caffeine (*p* < 0.001), whereas reported intakes of decaffeinated coffee and other caffeinated beverages showed no association. In ROC analysis, optimal discrimination between individuals consuming *less than one caffeinated coffee/week,* vs. *at least one coffee,* was obtained for 24-h urinary paraxanthine (Area Under Curve (AUC) = 0.868, 95% Confidence Interval (CI) [0.830;0.906]), with slightly lower performance for theophylline and caffeine, whereas theobromine did not allow any discrimination.

**Conclusion:**

Our results suggest that reported consumption of caffeinated coffee is positively associated with 24-h urinary excretion of caffeine, paraxanthine, and theophylline, and may be used as a marker of caffeine intake for epidemiological studies.

**Electronic supplementary material:**

The online version of this article (doi:10.1186/s12986-016-0144-4) contains supplementary material, which is available to authorized users.

## Background

Coffee is one of the most widely consumed beverages in the world and the source of more than 1000 biologically active compounds [1] such as alkaloids, polyphenols, diterpene alcohols and others. The most abundant biologically active molecule in coffee is caffeine, a purine alkaloid, which is also found in soft drinks, tea and numerous other food items [2, 3]. More than 70% of caffeine is provided by caffeinated coffee consumption, and then metabolized by the liver CYP1A2 enzyme into paraxanthine (~80%), theobromine (~12%) and theophylline (~4%) [4]. Caffeine and caffeine related metabolites belong to the group of methylxanthines: a family of nonspecific adenosine receptor antagonists with several physiological properties, including diuresis and natriuresis [5, 6]. Due to caffeine’s virtual omnipresence in human diet, the health consequences of coffee and caffeine consumption are of major interest. While acute coffee and caffeine intake activate sympathomimetic effects such as increased blood pressure [7] and lipolysis [8], recent epidemiological studies suggested that chronic coffee and caffeine intake may exert beneficial long-term health effects by reducing the risk of chronic diseases such as type 2 diabetes, cardiovascular disease (CVD), some types of cancer [9], and even mortality [10, 11].

A key step in understanding coffee- or caffeine-associated health outcomes consists in accurately assessing individual’s exposure to these compounds. Population-based studies are mainly relying on questionnaires, which collect self-reported information on the quantity, frequency and the type of intake [12–14]. Although these questionnaires provide valuable epidemiological information, they remain approximate and subject to meaningful misclassification/measurement bias [15]. Validation by comparison with 24-h dietary recalls, daily diary records [16, 17] or 24-h excretion of specific biomarkers [18] are needed. Regarding caffeine intake, objective data are still lacking. Only a limited number of studies have compared self-reported consumption of different caffeinated beverages and caffeine with urinary excretion of caffeine metabolites [19] or with other biological material [20, 21], and no such associations have yet been investigated in large population-based studies.

In the present work, we compared self-reported consumption of coffee and other caffeinated beverages with 24-h urinary excretions of caffeine, a validated biological marker of caffeine intake [19, 22], and its metabolites in the Swiss Kidney Project on Genes in Hypertension (SKIPOGH) cohort. The SKIPOGH study is of particular interest regarding this association, as it extensively investigates both genetic and environmental determinants of blood pressure, including caffeine intake through self-report as well as 24-h urinary excretion measures [4].

## Methods

### Study population and design

We used data from the SKIPOGH project, a family and population-based cross-sectional study exploring genetic and environmental determinants of blood pressure. Participants were recruited from December 2009 until April 2013 in the Swiss cities of Lausanne, Geneva and Bern as previously described [23, 24]. Inclusion criteria were: (1) written informed consent; (2) minimum age of 18 years; (3) Caucasian origin, defined as having both parents and grandparents born in a restricted list of countries; (4) at least one, and preferably three, first-degree family members also willing to participate. Women who reported being pregnant were excluded from the SKIPOGH study. All included participants sustained a morning medical visit after an overnight fast, completed a self-administered life/medical history questionnaire and were asked to collect urine over 24-h. All participants signed written informed consent. The total study population included 1128 participants coming from 273 nuclear families.

### Clinical and biological data

Participants came for the study visit at one of the three medical centers, and filled in a standardized questionnaire at home. The questionnaire focused on a variety of issues including lifestyle habits as well as medical history. Body weight (kg), height (cm) and waist and hip circumferences (cm) were measured according to standard procedures. Body mass index (BMI) was defined as weight in kg divided by height in meters squared. Venous blood samples were drawn while fasting. Kidney function and other biological markers were measured in local university laboratories using standard clinical laboratory methods. The collection of 24-h urine sample was previously described [23, 25, 26]. Smoking status was categorized as current and noncurrent smokers, the latter category including never smokers and ex-smokers. Alcohol consumption was defined as consuming more than one alcoholic beverage per week (“Yes” or “No”).

### Caffeinated beverages frequency questionnaire

Caffeinated beverages frequency questionnaire, presented in the Appendix, was used to assess caffeine exposure through reported consumption of caffeinated beverages, and was based on a literature review of dietary questionnaires used in Europe as well as on cultural aspects of caffeinated beverage consumption in Switzerland [27–29]. The SKIPOGH questionnaire on caffeinated beverages was prospectively introduced in the second period of study recruitment and submitted to 657 participants (58%). We considered three major items; 1) caffeinated coffee, 2) decaffeinated coffee, and 3) other caffeinated beverages (soft drinks, energy drinks, black or green tea). For each item, consumption frequency was assessed through the question “How often do you consume caffeinated coffee/decaffeinated coffee/caffeinated beverages other than coffee?”), with five possible answers: “Never”; “1–4 beverages/month”, “1–4 beverages/week”, “≥5 beverages a week”, “≥1 beverage/day”. Moreover, individuals who reported consuming ≥1caffeinated coffee per day also reported the number of daily cups. The questionnaire also reported the time and quantity of the last beverage consumed before blood was drawn.

### Urinary caffeine metabolites

Caffeine (urine and plasma), paraxanthine (urine and plasma), theobromine (urine) and theophylline (urine) were quantified by ultra-high performance liquid chromatography (Waters ACQUITY UPLC I-Class for urine and Waters ACQUITY UPLC for plasma) coupled to electrospray ionization-tandem mass spectrometry (Waters Xevo TQ-S for urine and Waters TQD for plasma). Sample preparation was performed by simple dilution for urine and protein precipitation for plasma. Limit of quantification in urine was 10 ng/ml for caffeine, paraxanthine and theophylline and 20 ng/ml for theobromine, and in plasma was 5 ng/ml for caffeine and paraxanthine. The methods were fully validated according to the latest international guidelines using a stable isotope-labeled internal standard for each analyte. Expanded uncertainty (95% confidence level) calculated during routine use was 8.2, 7.6, 7.8 and 8.1% for caffeine, paraxanthine, theobromine and theophylline in urine, respectively, and 9.4 and 10.5% for caffeine and paraxanthine in plasma, respectively (Ansermot et al. manuscript in preparation, detailed method available on request).

### Statistical analyses

Continuous variables were described with median or mean and standard deviation. Categorical variables were described with percentages. Twenty-four hours urinary caffeine, paraxanthine, theophylline and theobromine were winsorised to exclude extreme outliers (99^th^ percentile) as performed previously [30–32]. We used quantile regression to explore the association between reported consumption frequency of caffeinated coffee, other caffeinated beverages and decaffeinated coffee, and caffeine metabolites, within a non-adjusted model and a model adjusted for major confounders. Variables included in the fully adjusted model as potential confounders were a priori considered, given their reported or potential influence on caffeine intake and urinary caffeine and paraxanthine excretion [14]. The following confounding variables were included: age, sex, BMI, Chronic Kidney Disease-Epidemiology Collaboration Formula (CKD-EPI) for glomerular filtration rate (GFR), as well as current smoking and alcohol use. Creatinine excretion per body weight (mg/kg/24-h), urinary volume (ml) and/or urinary flow (ml/min) were used as covariates in the fully adjusted model to account for the quality of urine collection. The full-model was also adjusted for center to take into account the potential clustering of caffeine metabolites excretion [14]. Familial correlations were taken into account for all analyses. Statistical significances for association were set at a *p*-value <0.05. To further quantify the degree of association between reported consumption frequency of the three types of beverages and caffeine-derived urinary metabolites, we also performed a spearman correlation (*ρ*) analysis for the unadjusted model. All statistical analyses were conducted using STATA 14.0 (Stata Corp, Stata College Station, Texas, USA).

### Receiver operator characteristic analysis

To further explore the association between self-reported consumption frequency and 24-h urinary metabolites, we performed a Receiver Operator Characteristic (ROC) analysis between dichotomized consumption frequency of caffeinated coffee and 24-h urinary caffeine, paraxanthine, theophylline and theobromine. The ROC analyses were performed whenever the quantitative regression between self-reported consumption frequency and 24-h urinary excretion was significant. The dichotomized threshold based on the self-reported consumption frequency was defined as following: *Less than one caffeinated coffee per week* : “Never”, “1–4×/month”; *At least one caffeinated coffee per week*: “1–4×/week”, “≥5×/week”, “≥1×/day”. This threshold was chosen based on the results from preliminary ROC analysis of all different possible dichotomous thresholds.

We computed 95% confidence intervals (CI) for Area Under Curves (AUC) for the 24-h urinary caffeine, paraxanthine, theophylline and theobromine. Optimal sensitivity and specificity values were determined according to Youden index in ROC analysis [33].

## Results

Out of the 657 SKIPOGH participants who completed the caffeine beverages questionnaire, 598 participants (48% men) had no missing data on beverage frequency intake, 24-h metabolite excretion as well as other covariates, and were included in the study. Participants who were included in the analysis tended to be younger, had a slightly higher alcohol intake and were mainly recruited in Geneva and Lausanne.

We summarize the main characteristics of the sample according to sex in Table 1. Women had a lower BMI, a lower 24-h urinary creatinine, a lower 24-h urinary paraxanthine excretion, were less frequently smokers, consumed less frequently one or more alcoholic drink per week than men. Men and women also had different consumption patterns for decaffeinated coffee. Urinary paraxanthine and theobromine excretions were higher (more than 5 fold) than caffeine and theophylline in both sex.

**Table 1:** Baseline characteristics of participants included in the study (N = 598), SKIPOGH study (Switzerland, 2009–2013)

	Men (*n* = 288)	Women (*n* = 310)	*P*-value^a, b^
Age, mean (SD)	46.16 (17.41)	46.17 (17.28)	0.972
BMI (kg/m2), mean (SD)	25.84 (4.1)	24.08 (4.56)	<0.001
Glomerular filtration rate, mean (SD)	97.46 (19.1)	95.68 (17.43)	0.116
Urinary parameters			
Urinary volume (ml/24-h), mean (SD)	1751.38 (801.87)	1687.39 (682.89)	0.635
Urinary flow (ml/min), mean (SD)	1.23 (0.57)	1.19 (0.5)	0.663
Urinary creatinine (mg/kg/24-h), mean (SD)	22.33 (5.46)	18.35 (4.38)	<0.001
Urinary caffeine (mg/24-h), median (IQR)	2.76 (3.61)	2.85 (4.03)	0.891
Urinary paraxanthine (mg/24-h), median (IQR)	11.33 (11.49)	9.43 (9.44)	<0.001
Urinary theobromine (mg/24-h), median (IQR)	11.63 (12.78)	10.9 (11.95)	0.212
Urinary theophylline (mg/24-h), median (IQR)	0.95 (1.09)	0.89 (0.96)	0.347
Study center, n (%)			0.805
Lausanne	83 (29%)	97 (31%)	
Geneva	101 (35%)	105 (34%)	
Bern	104 (36%)	108 (35%)	
Smoking, n (%)			0.024
No	212 (74%)	252 (81%)	
Yes	76 (26%)	58 (19%)	
Alcohol consumption, n (%)			<0.001
No	62 (22%)	135 (44%)	
Yes	226 (78%)	175 (56%)	
Caffeinated coffee consumption			0.219
Never	23 (8%)	41 (13%)	
1–4 times/month	15 (5%)	21 (7%)	
1–4 times/week	18 (6%)	22 (7%)	
≥ 5 times/week	11 (4%)	11 (4%)	
≥ 1 time/day	221 (77%)	215 (69%)	
Other caffeinated drink consumption, n (%)			0.755
Never	53 (18%)	56 (18%)	
1–4 times/month	76 (26%)	88 (28%)	
1–4 times/week	65 (23%)	59 (19%)	
≥ 5 times/week	16 (6%)	14 (5%)	
≥ 1 time/day	78 (27%)	93 (30%)	
Decaffeinated coffee consumption, n (%)			0.004
Never	233 (81%)	210 (68%)	
1–4 times/month	31 (11%)	62 (20%)	
1–4 times/week	8 (3%)	15 (5%)	
≥ 5 times/week	2 (1%)	6 (2%)	
≥ 1 time/day	14 (5%)	17 (5%)	

Data are mean (SD) or median (IQR) for continuous variables and N (%) for categorical variables

^a^Mann–Whitney *U* test was performed between men and women for continuous variables

^b^Chi2 contingency test was performed between men and women for categorical variables

In Table 2 we show the adjusted medians of 24-h urinary caffeine, paraxanthine, theophylline and theobromine (winsorised 99^th^ percentile), per consumption frequency of caffeinated coffee, other caffeinated beverages, and decaffeinated coffee for unadjusted and fully-adjusted quantile regression models. We observed a positive dose–response association between caffeinated coffee consumption frequency and urinary caffeine (*p*-value for trend <0.001, *ρ* = 0.473, *p*-value for *ρ* <0.001), paraxanthine (*p*-value for trend <0.001, *ρ* = 0.528, *p*-value for *ρ* <0.001) and theophylline (*p*-value for trend <0.001, *ρ* = 0.519, *p*-value for *ρ* <0.001) in the unadjusted and the fully adjusted models. We did not observe any significant association between consumption frequencies of other caffeinated beverages or decaffeinated coffee with any of the caffeine derived metabolites.

**Table 2:** Adjusted medians for 24-h excreted urinary metabolites [mg] according to the consumption frequencies of caffeinated coffee, other caffeinated beverages and decaffeinated coffee (quantile regression)

	Never	1–4×/Month	1–4×/Week	5×/Week	> = 1×/day	*P* for trend^a^	*ρ* ^c^	*P* ^d^
Adjusted medians for 24-h urinary caffeine [mg] (winsorised 99th percentile)								
Caffeinated coffee								
Unadjusted model	0.791	1.270	1.890	2.469	3.612	<0.001	0.473	<0.001
Fully adjusted model^b^	1.382	1.655	2.262	2.959	3.724	<0.001		
Other caffeinated drinks								
Unadjusted model	3.144	3.116	2.521	1.787	2.954	0.578	−0.069	0.090
Fully adjusted model^b^	3.419	3.285	3.034	1.899	3.268	0.702		
Decaffeinated coffee								
Unadjusted model	2.694	2.842	2.787	6.576	3.128	0.374	0.039	0.346
Fully adjusted model^b^	3.273	2.929	3.022	4.586	3.032	0.612		
Adjusted medians for 24-h urinary paraxanthine [mg] (win. 99th perc.)								
Caffeinated coffee								
Unadjusted model	2.331	4.269	6.833	6.923	12.512	<0.001	0.528	<0.001
Fully adjusted model^b^	3.847	4.287	7.174	8.755	12.456	<0.001		
Other caffeinated drinks								
Unadjusted model	12.361	9.993	8.706	8.361	10.706	0.469	−0.061	0.133
Fully adjusted model^b^	12.781	10.506	9.368	8.533	11.150	0.185		
Decaffeinated coffee								
Unadjusted model	9.875	9.571	9.500	17.852	11.407	0.324	0.057	0.164
Fully adjusted model^b^	10.498	10.271	10.188	11.503	11.829	0.494		
Adjusted medians for 24-h urinary theophylline [mg] (win. 99th perc.)								
Caffeinated coffee								
Unadjusted model	0.266	0.353	0.660	0.689	1.163	<0.001	0.519	<0.001
Fully adjusted model^b^	0.347	0.396	0.603	0.835	1.145	<0.001		
Other caffeinated drinks								
Unadjusted model	1.036	1.007	0.734	0.724	0.935	0.093	−0.075	0.069
Fully adjusted model^b^	1.100	0.982	0.842	0.684	0.965	0.519		
Decaffeinated coffee								
Unadjusted model	0.870	1.034	0.683	1.406	1.081	0.155	0.040	0.329
Fully adjusted model^b^	0.933	0.901	0.749	1.563	0.923	0.972		
Adjusted medians for 24-h urinary theobromine [mg] (win. 99th perc.)								
Caffeinated coffee								
Unadjusted model	11.352	12.911	13.743	11.747	10.977	0.398	−0.019	0.637
Fully adjusted model^b^	10.361	11.231	13.992	11.286	11.949	0.726		
Other caffeinated drinks								
Unadjusted model	11.900	10.287	11.456	10.904	11.623	0.870	0.013	0.746
Fully adjusted model^b^	13.256	11.203	10.854	9.342	13.351	0.472		
Decaffeinated coffee								
Unadjusted model	10.889	12.125	11.773	11.097	12.369	0.445	0.045	0.271
Fully adjusted model^b^	11.588	12.635	11.056	9.516	14.604	0.178		

^a^ *P*-value for linear trend (Reported consumption frequency: lowest vs. highest)

^b^Model was adjusted for age, sex, BMI, urinary creatinine, glomerular filtration rate, urinary volume, urinary flow, study center, smoking and alcohol status

^c^Spearman correlation coefficient (*ρ*) for the association between self-reported consumption frequency and 24-h urinary excretion

^d^Spearman correlation coefficient associated *p*-value

**Fig. 1:**
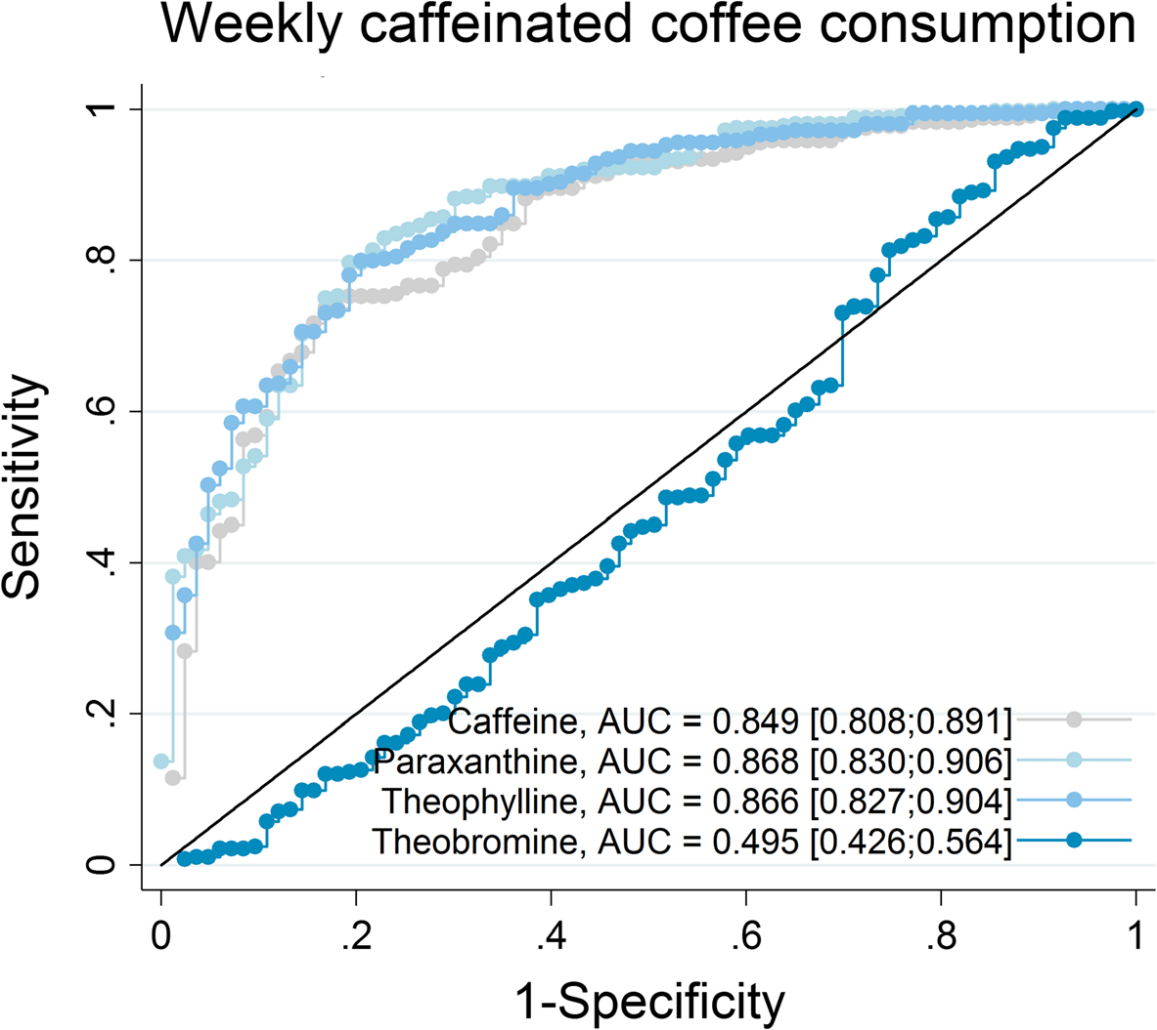
Receiver Operator Characteristic (ROC) curves for weekly dichotomized self-reported caffeinated coffee consumption, based on 24-h urinary caffeine, paraxanthine, theophylline, theobromine (win: 99^th^ percentile winsorised). Sensitivity, specificity and cutoff points are reported in Table 3. AUC: Area under curve [95% CI]

In Fig. 1, we present ROC analysis results, including AUC for the dichotomized caffeinated coffee consumption frequency based on 24-h urinary excretions of caffeine, paraxanthine, theophylline, and theobromine, whereas related optimal cutoff and sensitivity/specificity values are presented in Table 3. Optimal discrimination between individuals who consumed *less than one caffeinated coffee per week* versus *at least one caffeinated coffee*, was obtained based on 24-h urinary paraxanthine AUC = 0.868, 95% CI [0.830;0.906] with an optimal cutoff at 2.582 mg, followed by theophylline AUC = 0.866, 95% CI [0.827;0.904] (0.774 mg), and caffeine AUC = 0.849, 95% CI [0.808;0.891] (1.391 mg). Regarding theobromine, AUC was 0.495, 95% CI [0.426;0.564], suggesting no discrimination power.

**Table 3:** Optimal sensitivity, specificity and cutoff points for weekly caffeinated coffee consumption based on 24-h urinary metabolites – ROC analysis (Fig. 1)

	Sensitivity	Specificity	Cutoff (mg)
24-h urinary metabolite			
24-h urinary caffeine	0.723	0.840	1.391
24-h urinary paraxanthine	0.801	0.790	2.582
24-h urinary theophylline	0.787	0.800	0.774
24-h urinary theobromine	0.938	0.160	1.665

To account for the fact that most of the participants simultaneously consumed different types of caffeinated beverages, we report in Additional file 1: Tables S1–S3 two-by-two combinations of consumption frequencies of caffeinated coffee, decaffeinated coffee and other caffeinated beverages. Overall, we observed that consumption of one type of beverage was generally associated with the consumption of another beverage (*p*-value < 0.05). Therefore, in order to examine 24-h metabolite excretion resulting from the exclusive consumption of only one type of beverage, we show in supplementary Additional file 1: Tables S4-S5 median 24-h urinary excretions of caffeine, paraxanthine, theophylline and theobromine by consumption frequencies of either caffeinated coffee or other caffeinated beverages, while the consumption frequency of the remaining beverage and decaffeinated coffee was “Never”. In Additional file 1: Table S4, despite an extremely heterogeneous group size according to different consumption frequencies, we observed an almost step-wise increase in 24-h urinary excretion of caffeine, paraxanthine and theophylline as the consumption frequency of caffeinated coffee increased, which is in line with results presented in Table 2. In Additional file 1: Table S5, we also observed an increasing 24-h urinary excretion of all four caffeine derived metabolites as the consumption frequency of other caffeinated beverages increased.

### Discussion

In this population-based Swiss study, we found a strong association between reported consumption of caffeinated coffee and 24-h urinary excretion of paraxanthine, theophylline and caffeine, which is in line with previous research [19–22]. Our results suggest that self-reported consumption with the question “How often do you consume caffeinated coffee?”- could be used as a proxy of caffeine exposure, if no caffeine derived metabolites are available. The information gathered from self-reported consumption of caffeine intake is reliable enough to highlight associations between caffeine intake and major phenotypes or outcomes [10, 24, 34]. With the exception of theobromine, ROC analysis also showed that the 24-h urinary excretions of paraxanthine, theophylline and caffeine discriminated well weekly self-reported consumption frequency of caffeinated coffee.

Regarding the association between caffeinated coffee intake and urinary excretion of caffeine derived metabolites, our results are in line with previous studies that tested the use of caffeine derived metabolites in urine, serum or umbilical cord blood, as a potential marker of self-reported caffeine intake [19–21]. While there were important methodological differences in terms of assessment of self-reported caffeine intake, metabolite measurements or population characteristics between our study and this previous research, the correlation coefficients for the association between caffeinated coffee intake *or* self-reported intake of total caffeine [19–21] and 24-h urinary excretion caffeine, paraxanthine and theophylline, were generally in the same order of magnitude, ranging between 0.4 and 0.6. Thus, the adjusted medians of urinary caffeine, paraxanthine, and theophylline were the lowest among participants reporting never consuming caffeinated coffee and the highest among participants reporting the highest intake of this beverage. These results are in line with previous research showing that caffeinated coffee constitutes the main source of measured caffeine and caffeine metabolites in both urine and serum [24, 35]. Theobromine levels did not differ across intake, which might be due to the fact that theobromine is mostly found in chocolate [36].

Conversely, consumption frequencies of other caffeinated beverages and decaffeinated coffee were not associated with urinary excretions of caffeine or any of the other caffeine metabolites. Of note, the median levels of caffeine, paraxanthine, and theophylline were higher among participants who reported “never other caffeinated drinks” or “never decaffeinated coffee” compared to participants who reported “never caffeinated coffee”. This can be largely explained by the fact that the majority of participants in the “never other caffeinated drinks” or “never decaffeinated coffee” groups reported very frequent caffeinated coffee intake (Additional file 1: Tables S1–S3). Therefore, the absence of association in Table 2 between consumption frequency of other caffeinated beverages and 24-h urinary caffeine, paraxanthine and theophylline, is likely due to the caffeine input of caffeinated coffee consumption, which may mask the caffeine input from other caffeinated beverages only and therefore affect the 24-h urinary metabolite excretion trend. Thus, once caffeine input by caffeinated coffee and decaffeinated coffee is excluded (Additional file 1: Table S5), a clear positive trend is observed between increasing frequency of other caffeinated beverages and 24-h urinary excretion of all four metabolites. We may assume that a positive trend might also be observed for an increasing consumption frequency of decaffeinated coffee as this beverage also contains a certain amount of caffeine [37], yet due to the total lack of participants for several consumption frequencies of exclusive decaffeinated coffee intake, this couldn’t be assessed here.

Regarding ROC analysis, the observed AUC values were in line with the results from quantitative regression. The strongest AUCs were observed for 24-h urinary paraxanthine, whereas there was no relation for 24-h urinary theobromine, supporting the fact that urinary paraxanthine is likely the most common metabolite of caffeine intake [38, 39]. Our results thus suggest that paraxanthine may be used as a gold standard in future analyses investigating the validity of coffee consumption based on other urinary metabolites.

### Strengths and limitations

This is, to our knowledge, the first study to investigate the association between consumption frequency of three different common drinks (caffeinated coffee, other caffeinated beverages, decaffeinated coffee) and the 24-h excretion of caffeine and three caffeine metabolites, in a population based study.

Our study has several limitations. First, the questionnaire on caffeine reflects local food habit. Previous investigations have suggested large national differences regarding consumption habits of caffeinated coffee, tea, soft drinks or energy drinks [40–43]. Consequently, our results might not be generalized to settings with different food habits. Second, the validity of 24-h urine excretion is known to depend on the quality of urine collection. We therefore adjusted our analyses for urinary creatinine and volume. Third, while we account for major potential confounders, residual confounding cannot be excluded and information on other potential confounders (e.g. CYP1A2 gene, liver function) was not available. Information on self-reported liver diseases, including malignant liver cancer, liver cirrhosis, chronic liver disease or unspecified liver disorders, was collected but none of the included participants reported any of the four liver-related disorders. Furthermore, we must also take into account that the questionnaire used here is not a 24-h dietary recall or 3 days-diet diary, it may therefore introduce bias because of the recalling abilities of the participants. Fourth, the results of the present study are based on observational data, thus, an intervention or an experimental approach shall be considered in order to further explore and validate the association between caffeinated beverage intake and 24-h urinary excretion.

## Conclusion

Our results suggest that there is a strong association between reported consumption of caffeinated coffee and 24-h urinary caffeine metabolites. The associations between reported consumption of other caffeinated drinks/decaffeinated coffee and 24-h urinary excretions are less clear. Finally, urinary paraxanthine appeared to best discriminate individuals who consumed less than one caffeinated coffee per week versus individuals who consumed more.

## Additional file

Additional file 1: Table S1. Reported consumption frequencies of caffeinated and decaffeinated coffee N (%). **Table S2.** Reported consumption frequencies of other caffeinated beverages and decaffeinated coffee N (%). **Table S3.** Reported consumption frequencies of caffeinated coffee and other caffeinated beverages N (%). **Table S4.** Median 24-h urinary excretion according to caffeinated coffee consumption frequency, in participants who reported “Never” consuming other caffeinated beverages and “Never” consuming decaffeinated coffee. **Table S5.** Median 24-h urinary excretion according to other caffeinated beverage consumption frequency, in participants who reported “Never” consuming caffeinated coffee and “Never” consuming decaffeinated coffee. (DOCX 35 kb)

## Abbreviations

AUC: Area Under Curve
BMI: Body Mass Index
CI: Confidence Interval
CKD-EPI: Chronic kidney disease-epidemiology collaboration formula
CVD: Cardiovascular disease
GFR: Glomerular filtration rate
IQR: Inter quartile range
ROC: Receiver Operator Characteristic
SD: Standard deviation
SKIPOGH: Swiss Kidney Project on Genes in Hypertension

## References

[1] O'Keefe JH (2013). Effects of habitual coffee consumption on cardiometabolic disease, cardiovascular health, and all-cause mortality. J Am Coll Cardiol.

[2] Rostagno MA (2011). Fast and simultaneous determination of phenolic compounds and caffeine in teas, mate, instant coffee, soft drink and energetic drink by high-performance liquid chromatography using a fused-core column. Anal Chim Acta.

[3] Barone J and H Roberts. Human consumption of caffeine, in Caffeine. 1984, Springer. p. 59–73.

[4] Guessous I, et al. Associations of Ambulatory Blood Pressure With Urinary Caffeine and Caffeine Metabolite Excretions. Hypertension, 2014: p. HYPERTENSIONAHA. 114.04512. 10.1161/HYPERTENSIONAHA.114.04512 25489060

[5] Fredholm BB (2001). International Union of Pharmacology. XXV. Nomenclature and classification of adenosine receptors. Pharmacol Rev.

[6] WILCOX CS (1999). Natriuretic and diuretic actions of a highly selective adenosine A1 receptor antagonist. J Am Soc Nephrol.

[7] Nurminen ML (1999). Coffee, caffeine and blood pressure: a critical review. Eur J Clin Nutr.

[8] Fried RE (1992). The effect of filtered-coffee consumption on plasma lipid levels. Results of a randomized clinical trial. JAMA.

[9] Campos H, Baylin A (2007). Coffee consumption and risk of type 2 diabetes and heart disease. Nutr Rev.

[10] Freedman ND (2012). Association of coffee drinking with total and cause-specific mortality. New Engl J Med.

[11] Ding M, et al. Association of Coffee Consumption with Total and Cause-Specific Mortality in Three Large Prospective Cohorts. Circulation, 2015: p. CIRCULATIONAHA. 115.017341. 10.1161/CIRCULATIONAHA.115.017341 PMC4679527 26572796

[12] Willett WC (1985). Reproducibility and validity of a semiquantitative food frequency questionnaire. Am J Epidemiol.

[13] Rimm EB (1992). Reproducibility and validity of an expanded self-administered semiquantitative food frequency questionnaire among male health professionals. Am J Epidemiol.

[14] Guessous I (2015). Associations of ambulatory blood pressure with urinary caffeine and caffeine metabolite excretions. Hypertension.

[15] Bracken MB (2002). Heterogeneity in assessing self-reports of caffeine exposure: implications for studies of health effects. Epidemiology.

[16] Bolca S (2009). Validity and reproducibility of a self-administered semi-quantitative food-frequency questionnaire for estimating usual daily fat, fibre, alcohol, caffeine and theobromine intakes among Belgian post-menopausal women. Int J Environ Res Public Health.

[17] Schliep KC (2013). Validation of different instruments for caffeine measurement among premenopausal women in the BioCycle study. Am J Epidemiol.

[18] Brantsaeter A (2009). Evaluation of urinary iodine excretion as a biomarker for intake of milk and dairy products in pregnant women in the Norwegian Mother and Child Cohort Study (MoBa). Eur J Clin Nutr.

[19] Rybak ME (2015). Urine excretion of caffeine and select caffeine metabolites is common in the US population and associated with caffeine intake. J Nutr.

[20] Grosso LM (2008). Prenatal caffeine assessment: fetal and maternal biomarkers or self-reported intake?. Ann Epidemiol.

[21] Klebanoff MA (1998). Serum caffeine and paraxanthine as markers for reported caffeine intake in pregnancy. Ann Epidemiol.

[22] Crews HM, Olivier L, Wilson LA (2001). Urinary biomarkers for assessing dietary exposure to caffeine. Food Addit Contam.

[23] Pruijm M (2013). Heritability, determinants and reference values of renal length: a family-based population study. Eur Radiol.

[24] Guessous I (2012). Caffeine intake and CYP1A2 variants associated with high caffeine intake protect non-smokers from hypertension. Hum Mol Genet.

[25] Alwan H (2014). Epidemiology of masked and white-coat hypertension: the family-based SKIPOGH study. PLoS One.

[26] Ponte B (2014). Reference values and factors associated with renal resistive index in a family-based population study. Hypertension.

[27] Hercberg S (2010). The Nutrinet-Santé Study: a web-based prospective study on the relationship between nutrition and health and determinants of dietary patterns and nutritional status. BMC Public Health.

[28] AddictionSuisse. Factsheet Boissons énergisantes. 2011 [cited 2014; Available from: http://www.guide-ecole.ch/Htdocs/Files/v/8541.pdf/Guide/Alimentation/boissonsenergisantes/Factsheetenergydrinksf.pdf?download=1.

[29] ANSES, Boissons dites énergisantes : l’Anses met en garde contre des modes de consommation à risques. 2013, ANSES.(Accessed 2 Dec 2014).

[30] Ponte B. et al. Copeptin Is Associated with Kidney Length, Renal Function, and Prevalence of Simple Cysts in a Population-Based Study. Journal of the American Society of Nephrology: JASN, 2014. 10.1681/ASN.2014030260 PMC4446870 25270071

[31] Tukey JW. The Future of Data Analysis. Ann Math Stat. 1962;33:1–67.

[32] Liao J (2014). Impact of measurement error on testing genetic association with quantitative traits. PLoS One.

[33] Youden WJ (1950). Index for rating diagnostic tests. Cancer.

[34] Mitchell DC (2014). Beverage caffeine intakes in the U.S. Food Chem Toxicol.

[35] Heckman MA, Weil J, Gonzalez de Mejia E (2010). Caffeine (1, 3, 7-trimethylxanthine) in foods: a comprehensive review on consumption, functionality, safety, and regulatory matters. J Food Sci.

[36] Matissek R (1997). Evaluation of xanthine derivatives in chocolate–nutritional and chemical aspects. Zeitschrift für Lebensmitteluntersuchung und-Forschung A.

[37] McCusker RR (2006). Caffeine content of decaffeinated coffee. J Anal Toxicol.

[38] Guerreiro S (2008). Paraxanthine, the primary metabolite of caffeine, provides protection against dopaminergic cell death via stimulation of ryanodine receptor channels. Mol Pharmacol.

[39] Orrú M (2013). Psychostimulant pharmacological profile of paraxanthine, the main metabolite of caffeine in humans. Neuropharmacology.

[40] WorldResourcesInitiative. Current Worldwide Annual Coffee Consumption per capita. 2011. 12 Oct 2016. Available from: http://chartsbin.com/view/581.

[41] Barthel C, Wiegand S, Scharl S, et al. Patients’ perceptions on the impact of coffee consumption in inflammatory bowel disease: friend or foe? – a patient survey. Nut J. 2015;14:78. doi:10.1186/s12937-015-0070-8. 10.1186/s12937-015-0070-8 PMC4534065 26265051

[42] Basu S (2013). Relationship of soft drink consumption to global overweight, obesity, and diabetes: a cross-national analysis of 75 countries. Am J Public Health.

[43] Roberto A, Ferdman W, Euromonitor. Where the world’s biggest tea drinkers are. 2014. Available from: http://qz.com/168690/where-the-worlds-biggest-tea-drinkers-are/. Accessed 13 Oct 2016.

[44] Guessous I, Eap CB, Bochud M (2014). Blood pressure in relation to coffee and caffeine consumption. Curr Hypertens Rep.

