# Supplementary material for: Relation of 24-hour urinary caffeine and caffeine metabolite excretions with self-reported consumption of coffee and other caffeinated beverages in the general population
Source: Nutr Metab (Lond). 2016 Nov 17;13:81. doi: 10.1186/s12986-016-0144-4 (PMC5112879; doi:10.1186/s12986-016-0144-4)
Supplement: Additional file 1: Table S1. — Reported consumption frequencies of caffeinated and decaffeinated coffee N (%). Table S2. Reported consumption frequencies of other caffeinated beverages and decaffeinated coffee N (%). Table S3. Reported consumption frequencies of caffeinated coffee and other caffeinated beverages N (%). Table S4. Median 24-h urinary excretion according to caffeinated coffee consumption frequency, in participants who reported “Never” consuming other caffeinated beverages and “Never” consuming decaffeinated coffee. Table S5. Median 24-h urinary excretion according to other caffeinated beverage consumption frequency, in participants who reported “Never” consuming caffeinated coffee and “Never” consuming decaffeinated coffee. (DOCX 35 kb) [file 12986_2016_144_MOESM1_ESM.docx]

**Relation of 24-hour urinary caffeine and caffeine metabolite excretions with self-reported consumption of coffee and other caffeinated beverages in the general population**

Dusan Petrovic ^1^, Sandrine Estoppey Younes ^1^, [M](http://www.ncbi.nlm.nih.gov/pubmed/?term=Pruijm%20M%5BAuthor%5D&cauthor=true&cauthor_uid=25489060)enno Pruijm ^2^, [B](http://www.ncbi.nlm.nih.gov/pubmed/?term=Ponte%20B%5BAuthor%5D&cauthor=true&cauthor_uid=25489060)elén Ponte ^3^, [D](http://www.ncbi.nlm.nih.gov/pubmed/?term=Ackermann%20D%5BAuthor%5D&cauthor=true&cauthor_uid=25489060)aniel Ackermann ^4^, [G](http://www.ncbi.nlm.nih.gov/pubmed/?term=Ehret%20G%5BAuthor%5D&cauthor=true&cauthor_uid=25489060)eorg Ehret ^5^, [N](http://www.ncbi.nlm.nih.gov/pubmed/?term=Ansermot%20N%5BAuthor%5D&cauthor=true&cauthor_uid=25489060)icolas Ansermot ^6^, [M](http://www.ncbi.nlm.nih.gov/pubmed/?term=Mohaupt%20M%5BAuthor%5D&cauthor=true&cauthor_uid=25489060)arkus Mohaupt ^3^,  Fred Paccaud ^1^, [B](http://www.ncbi.nlm.nih.gov/pubmed/?term=Vogt%20B%5BAuthor%5D&cauthor=true&cauthor_uid=25489060)runo Vogt ^4^,  [A](http://www.ncbi.nlm.nih.gov/pubmed/?term=Pech%C3%A8re-Berstchi%20A%5BAuthor%5D&cauthor=true&cauthor_uid=25489060)ntoinette Pechère-Bertschi ^3^, [Pierre-Y](http://www.ncbi.nlm.nih.gov/pubmed/?term=Martin%20PY%5BAuthor%5D&cauthor=true&cauthor_uid=25489060)ves Martin ^3^, [M](http://www.ncbi.nlm.nih.gov/pubmed/?term=Burnier%20M%5BAuthor%5D&cauthor=true&cauthor_uid=25489060)ichel Burnier ^2^, [Chin B](http://www.ncbi.nlm.nih.gov/pubmed/?term=Eap%20CB%5BAuthor%5D&cauthor=true&cauthor_uid=25489060). Eap ^6, 7^, [M](http://www.ncbi.nlm.nih.gov/pubmed/?term=Bochud%20M%5BAuthor%5D&cauthor=true&cauthor_uid=25489060)urielle Bochud ^1^, Idris Guessous^1, 8, 9, 10^

1. Institute of Social and Preventive Medicine (IUMSP), Lausanne University Hospital, Route de la corniche 10, 1010 Lausanne, Switzerland

2. Department of Nephrology and Hypertension, Lausanne University Hospital, Rue du Bugnon 17, 1011 Lausanne, Switzerland

3. Department of Nephrology and Hypertension, University Hospital of Geneva (HUG), Rue Gabrielle Perret-Gentil 4, 1205 Geneva, Switzerland

4. University Clinic for Nephrology, Hypertension and Clinical Pharmacology, Inselspital, Bern University Hospital and University of Bern, Freiburgstrasse 15, 3010 Bern, Switzerland.

5. Department of Cardiology, University Hospital of Geneva (HUG), Rue Gabrielle Perret-Gentil 4, 1205 Geneva, Switzerland

6. Unit of Pharmacogenetics and Clinical Psychopharmacology, Centre for Psychiatric Neuroscience, Department of Psychiatry, Lausanne University Hospital, Prilly, Switzerland

7. School of Pharmaceutical Sciences, University of Geneva, University of Lausanne, Geneva, Switzerland

8. Unit of Population Epidemiology, Division of Primary Care Medicine, Department of Community Medicine and Primary Care and Emergency Medicine, University Hospital of Geneva (HUG), Rue Gabrielle Perret-Gentil 4, 1205 Geneva, Switzerland

9. Department of Epidemiology, Rollins School of Public Health, Emory University, Atlanta, USA

10. Lausanne University Outpatient Clinic, Rue du Bugnon 44, 1011 Lausanne, Switzerland

*Correspondence:

Dr. Idris Guessous, Unit of Population Epidemiology, University Hospital of Geneva (HUG), Rue Gabrielle Perret-Gentil 4, 1205 Geneva, Switzerland

Tel. +41 22 305 58 61

Fax +41 22 305 58 65

e-mail: idris.guessous@hcuge.ch

**Table S1:** Reported consumption frequencies of caffeinated and decaffeinated coffee N (%)

|  |  | **Decaffeinated coffee** | | | | |
| --- | --- | --- | --- | --- | --- | --- |
| **Caffeinated coffee** |  | Never | 1-4 times/month | 1-4 times/week | ≥5 times/week | ≥1 time/day |
|  | Never | 63 (14%) | 1 (1%) | 0 (0%) | 0 (0%) | 0 (0%) |
|  | 1-4 times/month | 28 (6%) | 5 (5%) | 2 (9%) | 0 (0%) | 1 (3%) |
|  | 1-4 times/week | 26 (6%) | 10 (11%) | 3 (13%) | 1 (13%) | 0 (0%) |
|  | ≥5 times/week | 17 (4%) | 2 (2%) | 2 (9%) | 0 (0%) | 1 (3%) |
|  | ≥1 time/day | 309 (70%) | 75 (81%) | 16 (70%) | 7 (88%) | 29 (94%) |
|  |  |  |  |  |  |  |
|  | P-value (chi2) | **0.006^a^** |  |  |  |  |
|  | P-value (Fisher) | **0.006^b^** |  |  |  |  |

^a^ P-value was computed from chi2 contingency test between the two consumption frequencies

^b^ P-value was computed from Fisher’s exact test between the two consumption frequencies, that were recoded: Low (Never ; 1-4 times/month) and Medium-High (1-4 times/week; ≥1 time/day)

**Table S2:** Reported consumption frequencies of other caffeinated beverages and decaffeinated coffee N (%)

|  |  | **Decaffeinated coffee** | | | | |
| --- | --- | --- | --- | --- | --- | --- |
| **Other caffeinated beverages** |  | Never | 1-4 times/month | 1-4 times/week | ≥5 times/week | ≥1 time/day |
|  | Never | 80 (18%) | 12 (13%) | 2 (9%) | 1 (13%) | 14 (45%) |
|  | 1-4 times/month | 116 (26%) | 33 (35%) | 8 (35%) | 1 (13%) | 6 (19%) |
|  | 1-4 times/week | 95 (21%) | 19 (20%) | 5 (22%) | 2 (25%) | 3 (10%) |
|  | ≥5 times/week | 22 (5%) | 6 (6%) | 2 (9%) | 0 (0%) | 0 (0%) |
|  | ≥1 time/day | 130 (29%) | 23 (25%) | 6 (26%) | 4 (50%) | 8 (26%) |
|  |  |  |  |  |  |  |
|  | P-value (chi2) | **0.049^a^** |  |  |  |  |
|  | P-value (Fisher) | **0.347^b^** |  |  |  |  |

^a^ P-value was computed from chi2 contingency test between the two consumption frequencies

^b^ P-value was computed from Fisher’s exact test between the two consumption frequencies, that were recoded : Low (Never ; 1-4 times/month) and Medium-High (1-4 times/week; ≥1 time/day)

**Table S3:** Reported consumption frequencies of caffeinated coffee and other caffeinated beverages N (%)

|  |  | **Other caffeinated beverages** | | | | |
| --- | --- | --- | --- | --- | --- | --- |
| **Caffeinated coffee** |  | Never | 1-4 times/month | 1-4 times/week | ≥5 times/week | ≥1 time/day |
|  | Never | 4 (4%) | 13 (8%) | 16 (13%) | 5 (17%) | 26 (15%) |
|  | 1-4 times/month | 2 (2%) | 8 (5%) | 10 (8%) | 3 (10%) | 13 (8%) |
|  | 1-4 times/week | 1 (1%) | 14 (9%) | 11 (9%) | 4 (13%) | 10 (6%) |
|  | ≥5 times/week | 1 (1%) | 7 (4%) | 9 (7%) | 2 (7%) | 3 (2%) |
|  | ≥1 time/day | 101 (93%) | 122 (74%) | 78 (63%) | 16 (53%) | 119 (70%) |
|  |  |  |  |  |  |  |
|  | P-value (chi2) | **<0.001**^a^ |  |  |  |  |
|  | P-value (Fisher) | **<0.001^b^** |  |  |  |  |

^a^ P-value was computed from chi2 contingency test between the two consumption frequencies

^b^ P-value was computed from Fisher’s exact test between the two consumption frequencies, that were recoded : Low (Never ; 1-4 times/month) and Medium-High (1-4 times/week; ≥1 time/day)

**Table S4:** Median 24-hour urinary excretion according to caffeinated coffee consumption frequency, in participants who reported “Never” consuming other caffeinated beverages and “Never” consuming decaffeinated coffee

|  | **Caffeinated coffee consumption frequency** | | | | |
| --- | --- | --- | --- | --- | --- |
|  | Never | 1-4 times/month | 1-4 times/week | ≥5 times/week | ≥1 time/day |
| 24-h urinary caffeine [mg] | 0.27 | 1.09 | 1.68 |  | 3.37 |
| 24-h urinary paraxanthine [mg] | 1.19 | 1.15 | 2.98 |  | 13.71 |
| 24-h urinary theophylline [mg] | 0.11 | 0.11 | 0.3 |  | 1.16 |
| 24-h urinary theobromine [mg] | 5.25 | 22.87 | 5.2 |  | 12.41 |
| N | 4 | 1 | 1 | 0 | 74 |

**Table S5:** Median 24-hour urinary excretion according to other caffeinated beverage consumption frequency, in participants who reported “Never” consuming caffeinated coffee and “Never” consuming decaffeinated coffee

|  | **Other caffeinated beverage consumption frequency** | | | | |
| --- | --- | --- | --- | --- | --- |
|  | Never | 1-4 times/month | 1-4 times/week | ≥5 times/week | ≥1 time/day |
| 24-h urinary caffeine [mg] | 0.27 | 0.57 | 0.81 | 0.92 | 0.9 |
| 24-h urinary paraxanthine [mg] | 1.19 | 1 | 2.24 | 3.24 | 4.6 |
| 24-h urinary theophylline [mg] | 0.11 | 0.17 | 0.28 | 0.21 | 0.36 |
| 24-h urinary theobromine [mg] | 5.25 | 8.96 | 11.4 | 12.42 | 14.77 |
| N | 4 | 13 | 16 | 5 | 25 |
